# Automatic microscopic detection of mycobacteria in sputum: a proof-of-concept

**DOI:** 10.1038/s41598-018-29660-8

**Published:** 2018-07-27

**Authors:** D. Zingue, P. Weber, F. Soltani, D. Raoult, M. Drancourt

**Affiliations:** MEPHI, Aix Marseille Université, IRD, IHU Méditerranée Infection, Marseille, France

## Abstract

The laboratory diagnosis of lung mycobacterioses including tuberculosis comprises the microscopic examination of sputum smear after appropriate staining such as Ziehl-Neelsen staining to observe acid-fast bacilli. This standard procedure is operator-dependant and its sensitivity depends on the duration of observation. We developed and evaluated an operator-independent microscopic examination of sputum smears for the automated detection and enumeration of acid-fast bacilli using a ZEISS Axio Scan.Z1 microscope. The sensitivity, specificity, positive predictive value, negative predictive values and accuracy were calculated using standard formulations by comparison with standard microscopic examination. After in-house parameterization of the automatic microscope and counting software, the limit of detection evaluated by seeding negative sputa with *Mycobacterium bovis* BCG or *Mycobacterium tuberculosis* H37Rv (10^0^–10^5^ bacilli/mL) was of 10^2^ bacilli/mL of sputum with a 100% positivity rate. Then, the evaluation of 93 sputum specimens including 34 smear-positive and 59 smear-negative specimens yielded a sensitivity of 97.06% [84.67–99.93%], a specificity of 86.44% [73.01–92.78%]. Up to 100 smear slides could be stocked for reading in the microscope magazine and results are exportable into the laboratory information system. Based on these preliminary results, we are implanting this automatic protocol in the routine workflow so that only smears detected positive by automatic microscopy are confirmed by standard microscopic examination.

## Introduction

Life-threatening lung tuberculosis is of public health concern in several regions in the world, as the World Health Organization reported 10.4 million new cases and 1.4 million deaths in 2016 worldwide^1^. In all affected countries, the laboratory diagnosis of lung tuberculosis is one important component of the fight against lung tuberculosis this disease, by assisting the medical management of patients including the isolation of contagious patients^2^. For that purpose, microscopic observation of tubercle bacilli in sputum smears, invented more than 100 years ago^1,3^ remains the cornerstone of the laboratory diagnosis of lung tuberculosis, infirming or confirming the diagnosis and contributing to assess the contagiousness of the patient^4,5^. In some remote areas across low- and middle income countries, microscopic examination of sputum smears is the only tool available for the laboratory diagnosis of lung tuberculosis^6–8^. Microscopic examination is performed after appropriate staining of mycobacteria and the Ziehl-Neelsen (ZN) staining is used worldwide for that purpose, staining in purple red acid-fast bacilli^9^. However, it is an operator-dependant technique of diagnosis, requiring specifically trained personnel^2^. Indeed, it is a time-consuming technique as the observation of at least 100 microscopic fields for at least 15 minutes for experienced staff is recommended^10^ and fluorescence microscopy (FM) smears can be examined in a fraction (about 25%) of the time needed for ZN smears^11^. Following this recommendation results in the fact that one laboratory personnel can examine only a limited number of slides per day; and routine observation may not be optimum, resulting in a variable sensitivity of 60% up to 70% compared to culture^12,13^. Moreover, results of the observation are manually reported, exposing to the risk of misreporting^14^. In countries with a high prevalence of lung tuberculosis, it has been noted that the workload required of technicians lead to professional overload and fatigue, resulting in microscopy work of lower quality^15^. In recent years, many countries including resource-limited and high TB burden employ light-emitting diode fluorescence microscopy (LED-FM) especially in the laboratories with high workload^16^. LED-FM has been endorsed by the World Health Organization as an alternative of (ZN in a phased manner for a more rapid tuberculosis (TB) diagnosis^16^. It has a higher sensitivity but lower specificity than ZN smear microscopy^16,17^. Uptake of LED-FM has been slow due to the loss of specificity compared with ZN microscopy, also sensitivity of LED-FM is reduced in HIV-infected patients^18^. In summary, the most common staining techniques commonly used to detect acid-fast bacilli (AFB) are ZN, Kinyoun, and the fluorescent technique, auramine-rhodamine. Nevertheless, ZN, Kinyoun and LED-FM are still creating considerable workloads for laboratories with limited resources.

Therefore, developing an operator-independent, automated reading of stained sputum spears smears to ensure the reproducibility of the observations may be of value^19^. Accordingly, previous studies have been conducted to automatize the microscopic detection of mycobacteria in ZN-stained sputum smears or fluorescent auramine-stained smears^2,4,10,20–25^. The aims of automation were to speed-up the screening process to cope efficiently with large numbers of smears^4,20^, to improve sensitivity and to reduce reliance on technicians^4^. However, none of these automated techniques readily replaced the standard microscopic examination in routine laboratory work for AFB detection^26^.

In France, where our laboratory is operating, the prevalence of lung tuberculosis is low with 5.1 cases per 10^5^ inhabitants^27,28^. We thought that in this specific context, a first step of automated microscopic detection of acid-fast bacilli would complement standard microscopic detection by sorting negative smears, in order to spare valuable technical work time. We here present a proof-of-concept of this approach which may assist other laboratories in their decision to implement automated smear reading in their routine workflow.

## Results

### Limit of the detection by automated microscopy

The acquisition/analysis combination detects AFB in sputum inoculation at a concentration of 10^2^/mL and the detection is even more tangible with an inoculum of 10^3^/mL. For an inoculum of 10^1^/mL, there is no detection of AFB by the ZEISS Axio Scan.Z1 Digital Slide Scanner. Each of the five fields analyzed contained approximately 11,000 × 7,500 points.

### Performances of automated microscopy

Among 93 sputum smear slides read by standard microscopy, 34/93 (36.56%) were positive by standard microscopy examination; automated microscopy detected 97% (33/34) of these slides as positive in addition to eight slides which had not been detected by standard microscopy. The resulting set includes 295 critical negative images from 59 subjects suspected of having pulmonary tuberculosis but eventually diagnosed without lung disease caused by mycobacteria and 170 positive images from 34 positive sputum specimens. These data yielded the following calculated values for four above-mentioned performance parameters of Zeiss automated method: 97.06% [84.67–99.93%] for the sensitivity (Se) and 86.44% [73.01–92.78%] for the specificity (Sp). The accuracy was 90.32. The Youden index was 0.82, the Yule coefficient was 0.99, the X² (Chi square) value was 58.28 (p < 0.001) and area under the ROC curve (AUC) was 0.91 [0.83–0.96].

**Figure 1:**
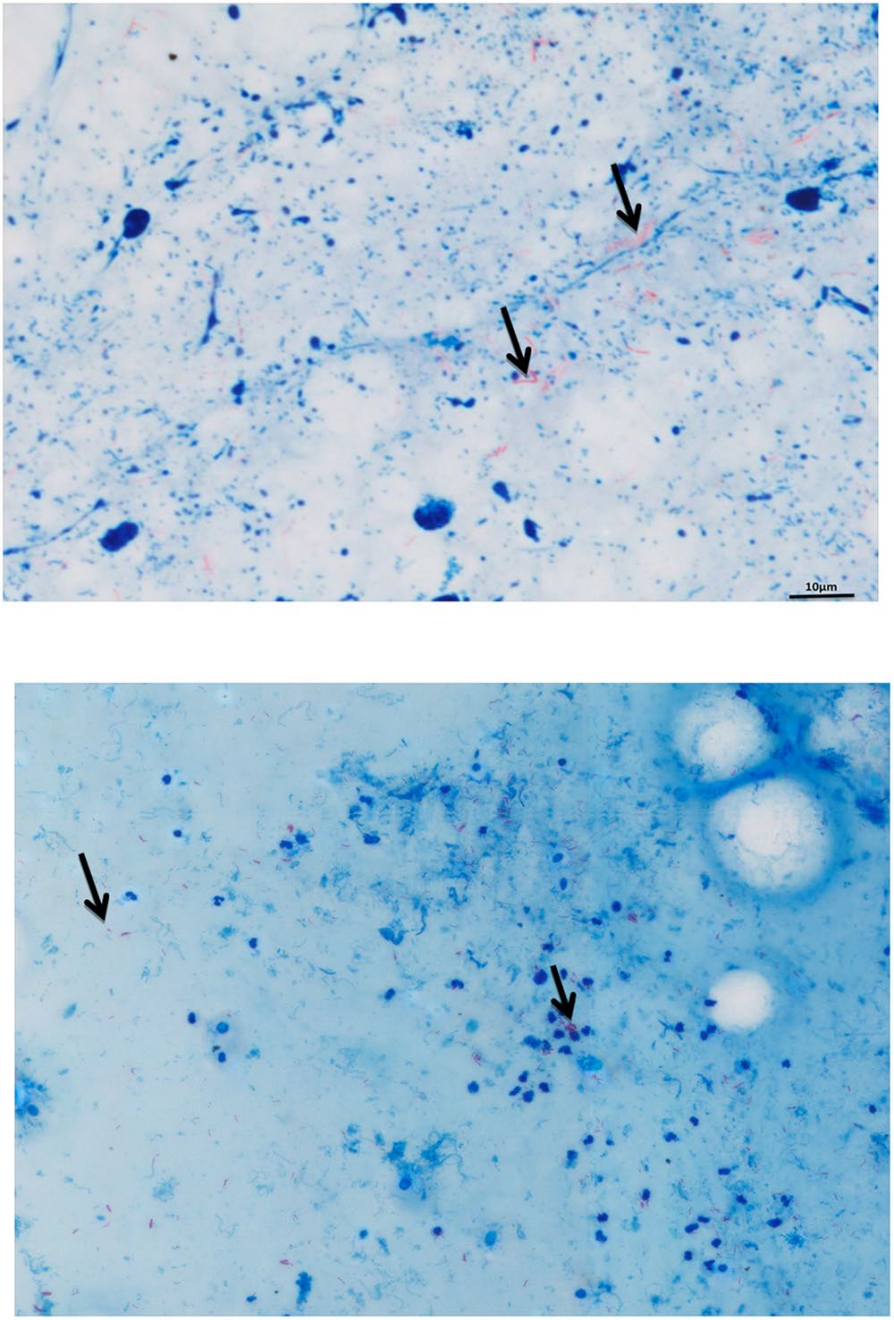
A *M. tuberculosis*-positive smear detected positive by standard light microscope (right) and the Zeiss Axio microscope and software here described (left). Black arrows point to *M. tuberculosis* organisms.

As for the 33 slides detected positive in common by standard and automated microscopy, numeration of AFB grading according to WHO and IUATLD recommendations yielded the same scale grade in 23/33 (68.6%) of slides. The types of images obtained from positive smears by the two methods are illustrated in Fig. 1. All 34 slides detected positive by standard microscopy (including the 33 slides also positive by automated microscopy) were culture-positive including 27 *M*. *tuberculosis* isolates, four *Mycobacterium intracellulare/chimeara* isolates and three *Mycobacterium simiae* complex isolates. Eight sputum specimens detected negative by standard microscopy and positive by automated microscopy remained sterile in culture. Further microscopic observation indicated that false-positivity was due to clusters of red dyes left on the slides after washing.

## Discussion

The Carl Zeiss Axio Scan.Z1 is used in many applications such as the pathogenesis of Alzheimer’s disease in neuroscience, cancer research, plant biology, developmental biology immunology toxicology as well as fluorescence *in situ* hybridization (FISH) and further applications^29–39^, but not yet for the detection of AFB. We developed an original protocol for the automated microscopic detection of AFB in sputum specimens with the aim of sorting negative smears and confirming only positive smears by standard microscopic examination. This preliminary work, which is a first, was done to demonstrate the feasibility of AFB detection by the Carl Zeiss Axio Scan.Z1 microscope and an important step towards a fully functional prototype that could be used in the context of large-scale studies such as national or regional tuberculosis surveys because the Carl Zeiss Axio Scan.Z1 microscope can analyze a hundred slides at the same time and can store images of virtual slides for future use. Automatic calibration, from geometry to color, accurately reproduces slides and makes them compatible between systems. We compared the two procedures of microscopy as the very same sputum specimens were prospectively examined in parallel by routine standard microscopic examination and the challenger automated microscopy procedure; with both analyses performed by independent operators.

Attempts to automate the reading of the slides after Ziehl-Neelsen staining were carried out with little success in routine use for the detection of mycobacteria^2,4,10,20–25^. Indeed, these studies were limited by the small number of samples. The present study of microscopy automation is the first of its kind to be successfully conducted on a large number of samples. We proceeded step by step in order to determine the minimum threshold of AFB in a sample required to have a positive result, then focused in a second step on achieving a negative predictive value as high as possible.

The automated microscopic detection here reported required a specific parameterization of the automated microscope and software in order to overcome unanticipated problems. In particular, we encountered problems when analyzing slides that had some black debris; and thick smears which retained dye clumps and staining artifacts; all initially read as AFB which yielded false positive results. These obstacles were overcome by focusing on the quality of smear preparation and careful washing of the slide; and repeated adjustments of the controller parameters to avoid debris counting. However, the algorithm used with Zeiss axio analyzes all the pixels in equal parts and makes it possible to detect all the bacilli present colored by Ziehl-Neelsen and to quantify them. ZEISS Axio Scan.Z1 Digital Slide Scanner, in addition to giving qualitative values for the diagnosis also allows quantifying the AFB, which is a parameter enabling us to monitor the effectiveness of the treatment. The detection of mycobacteria by the new algorithm proved to be an effective test with a Youden index close to 1.

False positive results were not problematic in our study as we decided that all the smears automatically detected as positive had to be confirmed by standard microscopic examination. One slide was falsely detected as negative by the automatic microscope, resulting from poor preparation of the slide and not from the automated reading of this slide. This data indicates that preparation of smear is crucial for automated detection, as it was previously reported for standard routine microscopy using Ziehl-Neelsen staining^40^.

Potential benefits of automated screening for mycobacterial lung disease are a rapid and accurate diagnosis, increased screening of the population, and reduced health risk to the staff processing slides^40^. When screening AFBs under an optical microscope at a convenient speed, a human observer may fail to observe bacilli, especially when the sample is paucibacillary, whereas one advantage of automated microscopy is its ability to read more than five microscopic fields per slide to increase the sensitivity. However, playback time increases simultaneously with the number of microscopic fields.

## Conclusions

In the specific context of a low prevalence of lung tuberculosis, automated microscopic detection of AFB using a Zeiss Axio Slide Scanner and home-adapted protocol for microscopy and software analyses achieved a high predictive negative value. This performance allowed us to pursue the on-going implementation of automated microscopic detection of AFB in the routine workflow of the laboratory to sort negative slides and control positive slides by standard microscopy. Additional advantages are a 100-slide hotel and digitalization of data for direct exporting into the laboratory information system, further optimizing workflow. We therefore propose that automated microscopic detection of AFB could already be used as a first-line microscopy diagnostic technique of lung tuberculosis in countries with a low prevalence of this disease. Further improvements in the specificity of the detection are required in order to progressively implement automated microscopy for the laboratory diagnosis of lung tuberculosis in countries where it is highly prevalent.

## Materials and Methods

### Clinical specimens

Sputum specimens collected as part of the routine diagnostic activity of our clinical microbiology laboratory (Institut Hospitalier Universitaire- Méditerranée Infection, Marseille, France) were prospectively included in this investigation. This study using anonymous, left-over routine specimens which have not been collected specifically for this study, received the agreement of the Institut Hospitalier Universitaire Méditerranée Infection, Marseille, France (CE 2016-025). Sputum specimens are manipulated in accordance with the relevant guideline and regulations. Sputum specimens were collected into a sterile, dry container and processed during 24–72 hours after collection. Microscopic detection of AFB was done as detailed below and chlorexidine-decontaminated sputum specimens were inoculated onto MOD9 culture medium as previously reported^41,42^. Colonies were detected by combining naked-eye detection and scanning detection as previously described^43^. Colonies were identified as *Mycobacterium tuberculosis* by using Biotyper Matrix Assisted Laser Desorption Ionization Time-of-Flight Mass Spectrometry (MALDI-TOF MS) (Bruker Daltonics, Bremen, Germany) and AutoXecute acquisition control in flexControl software version 3.0 and the identifications were obtained by MALDI Biotyper software version 3.0 with the Mycobacteria Library v2.0 (2014) database as previously described^44^.

### Microscopic examinations

As for microscopy, sputum were used to prepare duplicate smears per sample which were air dried, heat fixed and stained using a commercially-available kit featuring Kinyoun staining (kit Cold ZN, RAL, Toulouse, France). Standard microscopic observation was routinely done by the laboratory technicians using an Olympus BX40 light microscope (New York Microscope Co., USA) under oil immersion at x100 magnitude (camera of 640 × 480 pixels). For each sputum specimen, 100 microscopic fields are observed in routine^14^. Quantification of detected AFB was done according to international laboratory guidelines^14^. Automated microscopic detection was done using a Zeiss Axio Scan.Z1 Digital Slide Scanner (Carl Zeiss Microscopy, Marly-le-Roi, France). This automated microscope is featuring a 100-slide hotel, a LED light source, a Hitachi HV-F202SCL color camera with tri CCD 1,800 × 1,200 pixels, a plan apochromat 20×/0.8 objective comprising a 18,000 × 12,000-pixel microscopic field (0.22 μm/pixel). Axio Scan.Z1 employs fast filter wheels and can change channel in less than 50 milliseconds. Using 3-band and 4-band filter sets with Colibri.2 as a fluorescence light source, Axio Scan.Z1 makes millisecond-fast switches. The wavelength range is of 400 nm up to 700 nm and the resolution is of 10X (0.44 µm/pixel), 20X (0.22 µm/pixel) or 40X (0.11 µm/pixel). Using this resolution, a total of five fields were analyzed in each slide

Axio Scan.Z1 allows to scan samples and to create a virtual slide allowing the retrieval of a record of all virtual microscopy operations at the click of a button. The software module ZEN slidescan is capturing captures high volume quantitative image of 100 slides for 28 mm × 48 mm slides and the Scan time is of four minutes by slide. Each slide can be equipped with a barcode for recognition and archiving of the digitalized slides. Acquired images are saved in Jpeg or Tiff format and data could be accessed from anywhere by documenting and storing specimens as virtual slides, which could then be viewed on iPads with the free ZEN browser app. The results obtained by reading and counting the number of acid-fast bacilli found in a smear by the two methods of microscopy were graded according to the WHO and IUATLD^9^ recommendations.

### Parameterization of the zeiss slide scanner and software

The particular application of detecting AFB required a specific parameterization of the Zeiss Slide Scanner. MetaMorph® Microscopy Automation & Image Analysis Software (Molecular Devices Sunnyvale, California, USA) (https://www.moleculardevices.com/systems/metamorph-research-imaging/metamorph-microscopy-automation-and-image-analysis-software) were used for image treatment and AFB counting. Five fields were acquired per slide.

### Limit of detection by automated microscopy

In order to determine the limit of detection of AFB by using the Zeiss Slide Scanner, two AFB-negative sputum specimens were inoculated with either *Mycobacterium bovis* BCG strain Pasteur (Collection de l’Institut Pasteur, Paris, France) or with *Mycobacterium tuberculosis* H37Rv (Collection de l’Institut Pasteur) at inoculum concentration of 0, 10^0^, 10^1^,10^2^, 10^3^, 10^4^, 10^5^ mycobacteria/mL; then stained as described above and read by the Zeiss Slide Scanner without immersion oil.

### Performances of automated microscopy

The performances of automated microscopy were established by prospectively analyzing 34 positive AFB from 15 patients and 59 negative AFB sputum specimens collected as part of the routine diagnostic activity of the laboratory. The sampling was not exhaustive, however we used all positive slides obtained over the study period and added some negative sputum samples for the study. The sensitivity (Se) and specificity (Sp) were calculated by comparison with the results of standard microscopic examination of the same sputum specimens used as the reference method.

### Statistical analysis

The statistical software used was MedCalc Statistical Software Version 17.6 (MedCalc Software bvba, Ostend, Belgium). Performance of the method was estimated by the intrinsic characteristics of sensibility, specificity, prevalence and likelihood ratio. The confidence intervals of the percentage were calculated at the risk α at 0.05 of the binomial distribution and the performance results were expressed with IC95%.

### Ethical approval

This study using anonymous, left-over routine specimens which have not been collected specifically for this study, received the agreement of the Institut Hospitalier Universitaire Méditerranée Infection, Marseille, France (CE 2016-025).
